# Adaptive and Highly Efficient Thermoregulation Based on Multi‐Dimensional Janus Film

**DOI:** 10.1002/advs.76462

**Published:** 2026-07-09

**Authors:** Yuqing Shi, Yuting Fu, Xiong Yu, Yuao Guo, Yanjun Liu, Dan Luo, Yuanjing Lin

**Affiliations:** ^1^ School of Microelectronics Southern University of Science and Technology Shenzhen China; ^2^ Department of Applied Biology and Chemical Technology The Hong Kong Polytechnic University Hong Kong China; ^3^ Department of Electrical and Electronic Engineering Southern University of Science and Technology Shenzhen China; ^4^ State Key Laboratory of Quantum Functional Materials Southern University of Science and Technology Shenzhen China; ^5^ Energy Institute for Carbon Neutrality Southern University of Science and Technology Shenzhen China; ^6^ The Key Laboratory of the Third Generation Semi‐conductor Southern University of Science and Technology Shenzhen China

**Keywords:** janus film, radiative cooling, solar heating, sustainability, thermoregulation

## Abstract

Thermoregulation has attracted tremendous research attention as one of the eco‐friendly and sustainable strategies for temperature modulation. While most of the thermoregulation devices possess static cooling capabilities, the adaptability during nighttime or winter conditions is largely limited. Herein, we introduce a switchable dual‐mode thermoregulation system based on TiO_2_/MXene/PDMS (TMP) Janus film. Attributed to the strong solar scattering effect of TiO_2_ fillers and the intrinsically high mid‐infrared emissivity of the PDMS matrix, the TMP Janus film achieves a high solar reflectivity of 97% while maintaining a high MIR emissivity of 91% in cooling mode, and absorbs approximately 91% of solar irradiation in heating mode. By further integration with smart control modules, it allows autonomous switch between cooling and heating modes, which provides adaptive temperature regulation for practical applications. An ultra‐wide temperature regulation range with sub‐ambient cooling up to 17.89°C and above‐ambient heating up to 13.82°C were demonstrated. The proposed reversible thermoregulation Janus film offers a sustainable and effective strategy toward eco‐friendly and intelligent indoor temperature regulation.

## Graphical abstract

A switchable TiO_2_/MXene/PDMS Janus membrane enables dual‐mode radiative cooling and solar heating, achieving sub‐ambient cooling of 17.89°C and above‐ambient heating of 13.82°C. Adaptive temperature regulation was demonstrated with a low power integrated autonomous smart curtain system, providing a sustainable and energy‐efficient alternative for highly efficient thermal regulation.

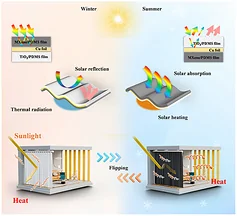

## 1 Introduction

Space thermoregulation plays a pivotal role in ensuring human comfort and promoting environmental sustainability, particularly in light of the substantial energy demands of traditional heating, ventilation, and air conditioning (HVAC) systems, which account for approximately 32%–33% of global energy consumption [1, 2, 3, 4]. Conventional thermoregulation devices typically employ static modes of either cooling or heating, which limit their adaptability across varying scenarios [5, 6, 7, 8]. Recent advances in adaptive thermal systems have shown promise for dynamic temperature control [9, 10, 11]. However, current technologies like thermochromic hydrogels and electrochromic devices encounter critical limitations, including insufficient heating efficiency, limited seasonal adaptability, and reliance on external power sources [12, 13, 14]. More importantly, a clear gap remains between material‐level thermal modulation and device‐level adaptive thermoregulation: most reported systems either focus on a single thermal function, require continuous external stimulation, or lack a scalable structure that can reversibly switch between cooling and heating in practical scenarios [15, 16, 17]. These challenges highlight the demand for innovative dual‐mode thermoregulation strategies that can passively adapt to both cooling and heating requirements.

To realize a high‐performance dual‐mode thermoregulation device, several critical factors need to be considered. Effective daytime radiative cooling requires high solar reflectivity and efficient mid‐infrared (MIR) emissivity within the atmospheric transmission window of 8–13 µm [18, 19, 20]. Dielectric materials such as titanium dioxide (TiO_2_) offer desirable optical scattering and stability [21, 22, 23, 24, 25]. Conversely, effective solar photothermal heating relies on materials with high solar absorption and efficient photothermal conversion capabilities [26, 27, 28, 29, 30], exemplified by two‐dimensional (2D) materials like MXene [31, 32, 33, 34, 35]. However, simply combining cooling and heating materials is insufficient, because their opposite optical requirements must be spatially separated, structurally integrated, and reversibly reconfigured without imposing continuous energy consumption [36]. Recently, Choi et al. reported a multimode adaptive thermoregulation system based on shape‐morphing porous thermoplastic polyurethane (TPU) combined with a photothermal layer, achieving a solar reflectance of 95.1% and an MIR emissivity of 88.5% in the 8–13 µm range. This work demonstrated an elegant strategy for adaptive thermal regulation through programmed shape transformation. However, further opportunities remain for developing structurally simple Janus architectures with spatially separated cooling and heating surfaces, direct integration into curtain‐like building components, and temperature‐feedback‐controlled autonomous switching for outdoor operation. An optimal dual‐mode design requires seamless integration of these contrasting functionalities in a scalable architecture, enabling smooth transitions between cooling and heating modes with minimal auxiliary energy input.

In this work, we present a novel switchable dual‐mode thermoregulation system based on a TiO_2_/MXene/PDMS (TMP) Janus film, shown in Figure 1. The innovation of this work lies in a mechanically reconfigurable asymmetric Janus architecture that integrates TiO_2_‐enhanced solar reflection, MXene‐enabled solar absorption, and PDMS‐derived MIR thermal emission within one scalable film. The TMP Janus film features a distinctive layered structure that consists of a TiO_2_‐doped PDMS cooling layer and an MXene‐doped PDMS heating layer, separated by an intermediate reflective copper (Cu) layer. Distinct from static thermoregulation coatings, the TMP Janus film achieves reversible cooling and heating through a mechanically switchable asymmetric architecture, while the integrated curtain prototype enables temperature‐feedback‐controlled mode switching with only low actuation energy. The film displays 97% solar reflectivity and 91% mid‐infrared absorptivity in cooling mode, while switching to 91% solar absorption in heating mode. Outdoor experiments validated a maximum sub‐ambient temperature reduction of 17.89°C and a maximum temperature rise of 10.47°C above ambient. This scalable TMP film system offers an effective and sustainable approach to passive indoor temperature regulation, demonstrating particular promise for smart building integration.

**FIGURE 1:**
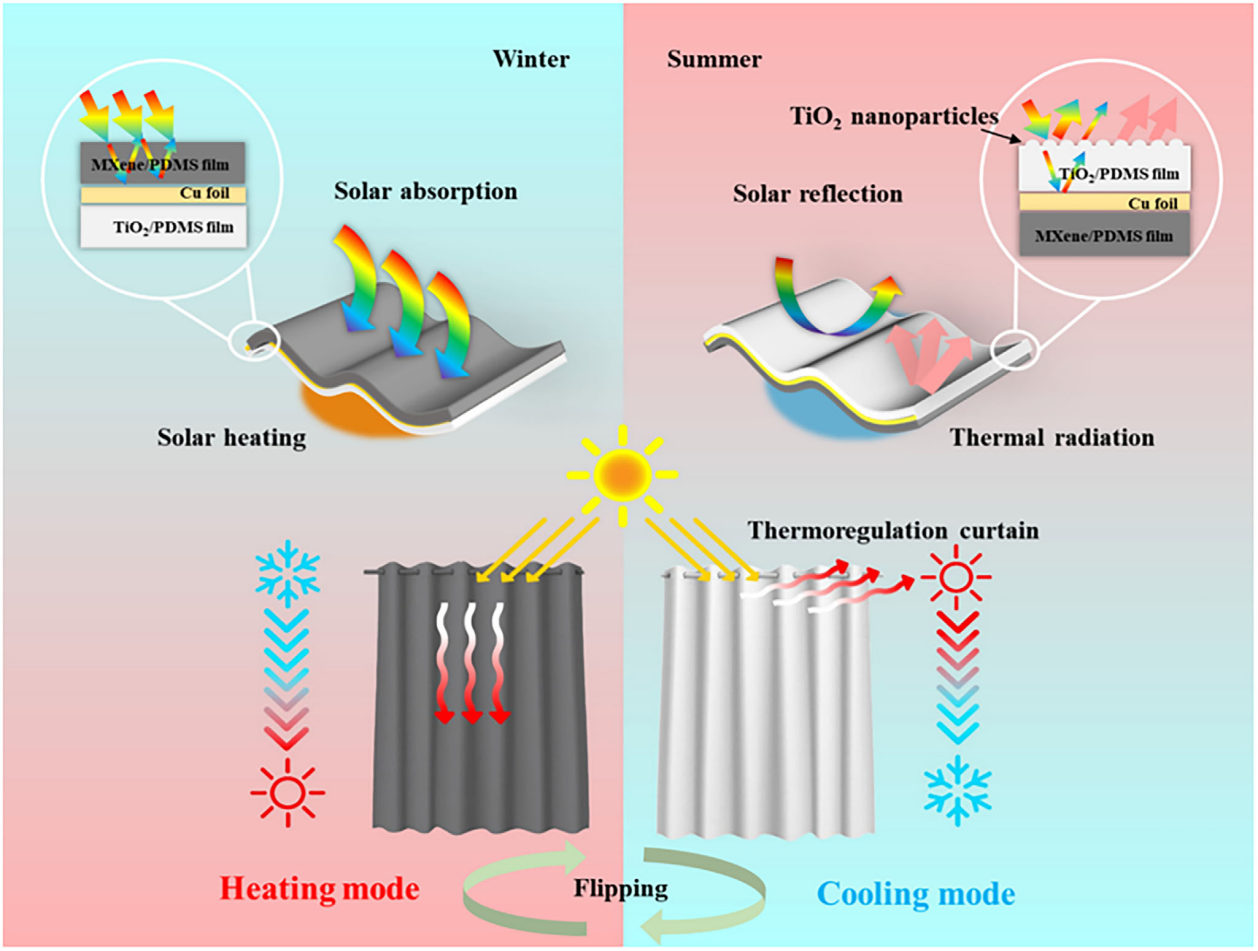
Schematic illustration of the TMP Janus film‐based dual‐functional smart curtain system that is capable of adaptive and switchable thermoregulation as demanded.

## 2 Results and Discussion

The TMP Janus film in sandwich structure was obtained by two step casting method, demonstrated in Figure 2a. The proposed TMP Janus film consists of three working layers, including cooling layer, middle Cu layer and heating layer. The cooling layer is constructed with a PDMS matrix embedded with TiO_2_ particles that serve as radiative cooling fillers. The heating layer is composed of PDMS doped with MXene particles for solar photothermal heating. A thin Cu interlayer, positioned between the two functional layers, acts as a physical separator, structural support, and reflective backing layer. This configuration prevents direct mixing between the TiO_2_/PDMS and MXene/PDMS layers while improving solar‐light management through optical back‐reflection. The cross‐sectional morphology of the film is shown in the SEM image in Figure 2ci, where all three layers can be clearly distinguished. Energy‐dispersive x‐ray spectroscopy (EDX) mapping presented in Figure 2cii–iv confirms the elemental distribution within the film. Silicon (Si), originating from the PDMS matrix, is uniformly present in both outer layers. Titanium (Ti) is primarily concentrated in the cooling layer, consistent with the incorporation of TiO_2_. In contrast, the heating layer contains fewer Ti elements due to the presence of MXene as the dominant filler.

**FIGURE 2:**
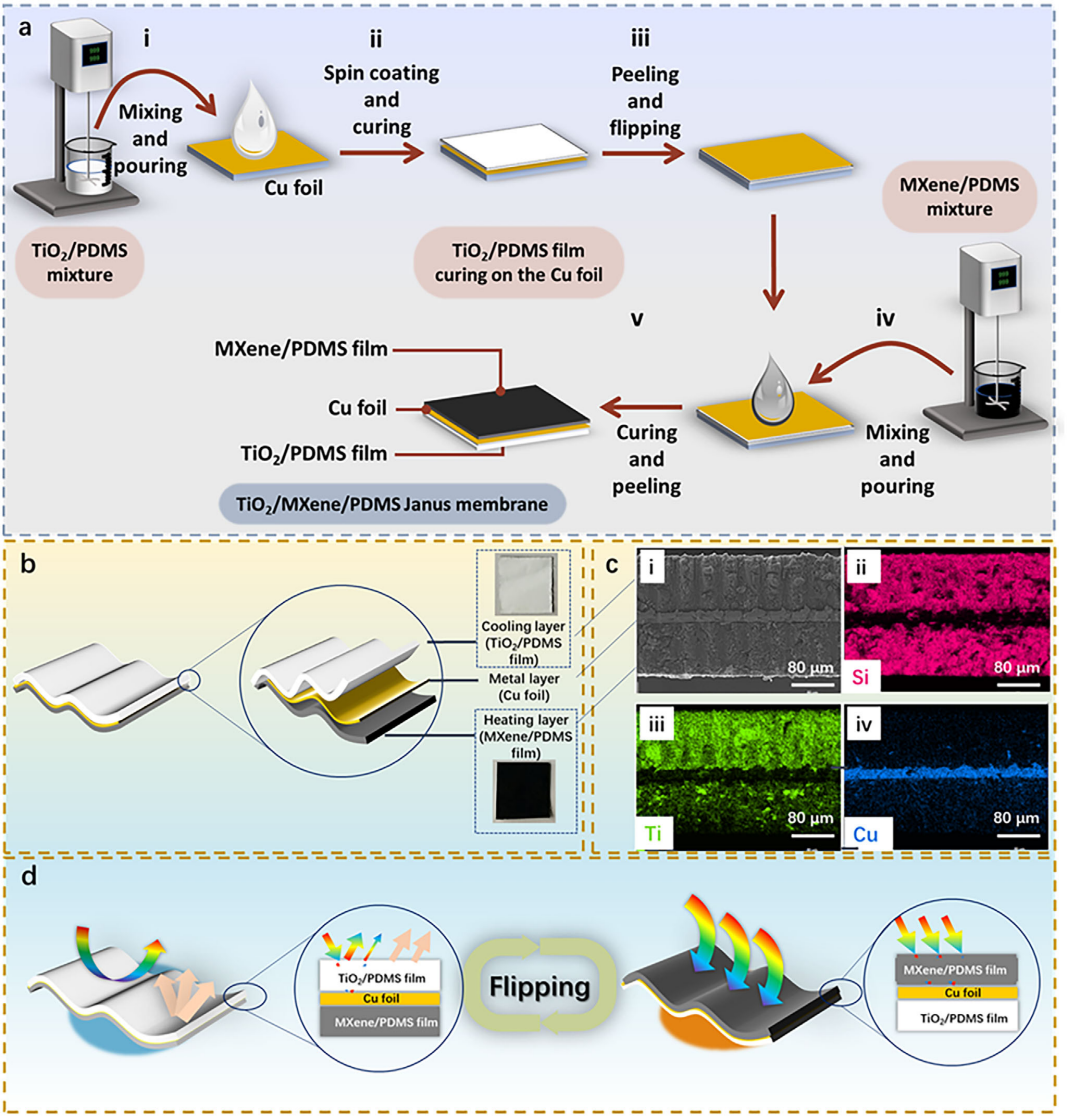
Fabrication process and structural characterization of TMP Janus film. (a) Schematic illustration of the two‐step casting process for the TiO_2_/MXene/PDMS Janus film. (b) Cross‐sectional schematic of the laminated sandwich structure, consisting of a cooling layer, middle Cu layer, and heating layer. (c) Cross‐sectional SEM image and corresponding EDX elemental mapping of Si, Ti, and Cu. (d) Illustration of the reversible thermoregulation mechanism under cooling and heating modes.

The reversible thermal behavior of the TMP Janus film is depicted in Figure 2d. When the cooling side faces upward, incident solar irradiation is strongly scattered by the TiO_2_‐loaded PDMS layer, while the residual transmitted light can be further reflected by the underlying Cu interlayer. This back‐reflection process improves solar rejection, particularly in the near‐infrared region. Conversely, when the heating side is oriented upward, the MXene particles absorb solar energy and convert it into heat, while the Cu interlayer can redirect unabsorbed light back toward the MXene/PDMS layer, thereby improving light utilization for photothermal heating.

To evaluate the influence of MXene film thickness on thermal properties, differential scanning calorimetry (DSC) was conducted on samples with varying thickness. As shown in Figure 3a, a distinct exothermic peak is observed near 40°C for all samples, which can be attributed to interfacial chain rearrangements between MXene nanosheets and the PDMS matrix upon heating [37, 38]. Negligible mass variation during TGA measurements indicates the thermal stability without decomposition or volatilization [39, 40, 41], as shown in Figure S1. These low‐temperature exothermic transitions with interfacial chain relaxations reported in polymer nanocomposites [42, 43]. The onset crystallization temperature (*T*_onset_), peak temperature (*T*_peak_), and enthalpy (*ΔH*) were quantified as Figure 3b,c. Among the tested samples, Sample 3 with 500 µm thickness exhibited the highest *T*_peak_ (47.66°C) and *ΔH* (92.62 J/g), suggesting optimized interfacial interactions that enhance thermal performance. This trend suggests intermediate thickness balances interfacial stress and defect density, facilitating efficient polymer‐filler reorganization [44, 45, 46, 47, 48]. The non‐monotonic *ΔH* variation aligns with established thickness‐dependent interface‐mediated relaxation behavior [49, 50]. Variations in internal stress, heat distribution, or filler dispersion during film formation may drive this effect. For example, 400 and 600 µm samples show transitional behavior where interfacial reorganization is suboptimal, suppressing enthalpy. The 500 µm film achieves a balance where interfacial contact and heat distribution synergize. Conversely, excessive thickness introduces kinetic barriers to crystallization, resulting in reduced *T*_peak_ and *ΔH*. These findings highlight that modulating MXene film thickness is critical to optimizing its thermal performance for applications requiring controlled phase transitions or energy storage.

**FIGURE 3:**
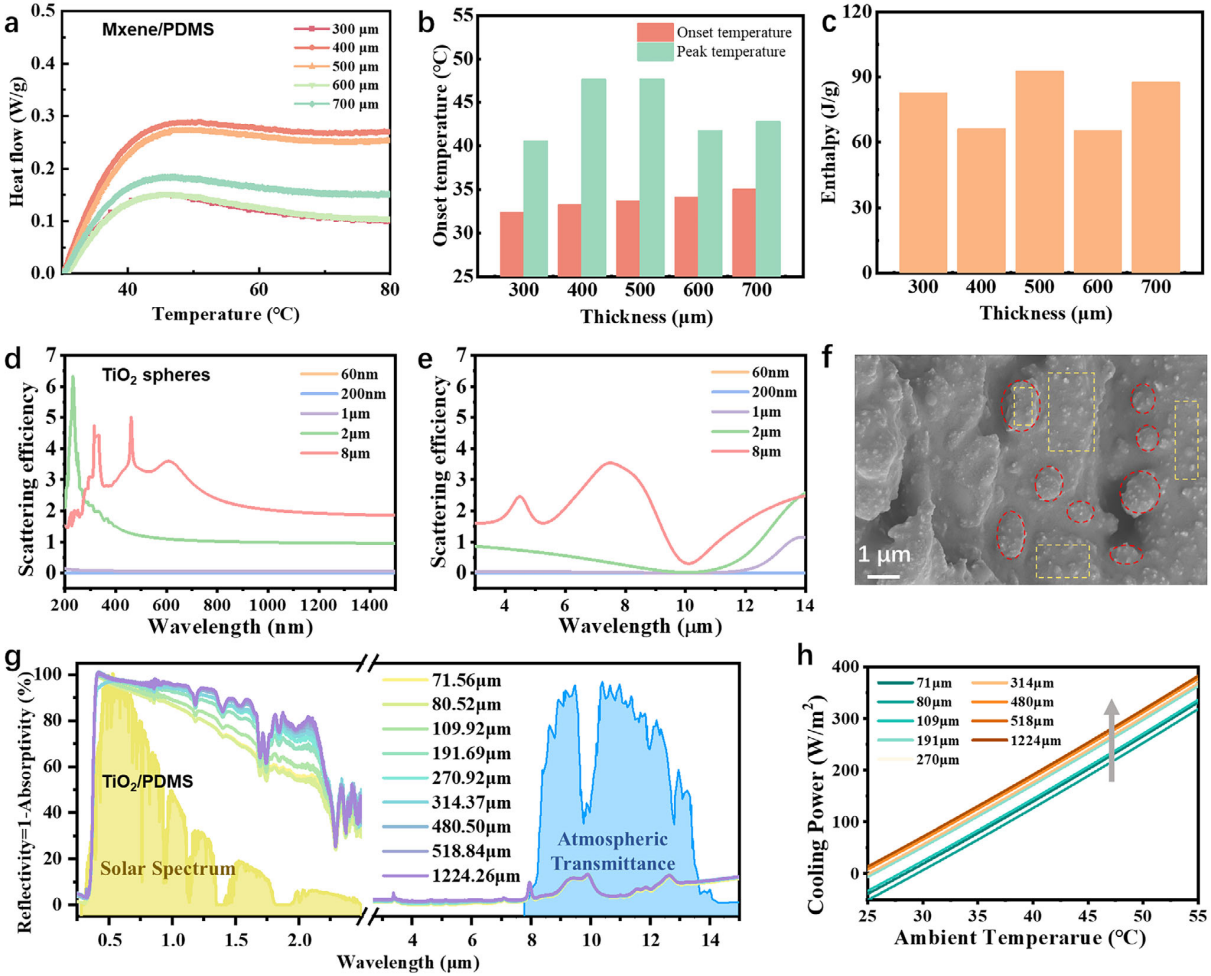
Optimization and characterization of MXene/PDMS heating layer and TiO_2_/PDMS cooling layers. (a) DSC curves for MXene/PDMS samples with film thicknesses of 300, 400, 500, 600, and 700 µm. (b) Onset and peak crystallization temperature as a function of MXene/PDMS film thickness. (c) Crystallization enthalpy values across different thicknesses. Simulated scattering efficiency of TiO_2_ spheres across the (d) solar (200–1500 nm) and (e) MIR (3–14 µm) spectra. (f) SEM images of the TiO_2_/PDMS film, showing 2 µm particles (red border) and surface‐attached nanoscale particles (yellow border). (g) Solar and MIR reflectivity of TiO_2_/PDMS film with varying thickness. (h) The cooling power of TiO_2_/PDMS film with different thickness.

Meanwhile, the scattering behavior of TiO_2_ particles was analyzed to guide the design of radiative cooling layers. According to Mie theory, the scattering efficiency of one specific material microsphere is strongly dependent on the particle size [33, 51, 52]. Simulations based on the radiative transfer equation and finite‐difference time‐domain (FDTD) methods (Figure 3d,e) reveal that larger TiO_2_ particles exhibit stronger scattering across both the solar and mid‐infrared (MIR) ranges [53, 54, 55]. To achieve high solar reflectivity and adequate MIR emissivity, particles with strong solar scattering and relatively weak MIR scattering are preferred. Although nanoscale TiO_2_ particles exhibit low MIR scattering, their efficiency in the solar spectrum is also limited. In contrast, 2 µm particles demonstrate high scattering in the solar region while maintaining moderate behavior in the MIR region, which is suitable for dual‐functional coatings.

Film thickness also plays a critical role in the radiative cooling property of TiO_2_/PDMS composites, as illustrated in Figure 3g. It is found that reflectivity increases with thickness, particularly in the near‐infrared range, and plateaus beyond approximately 518 µm. Across all samples, MIR absorptivity remains high (approximately 91%) and is not significantly affected by thickness. According to Kirchhoff's law, thermal emissivity equals optical absorptivity under thermal equilibrium, enabling prediction of radiative cooling power from absorptivity data. Figure 3h shows that cooling power increases with film thickness, reaching a maximum of 161.55 W/m^2^ at 40°C for the 518 µm film. Beyond this thickness, the enhancement becomes negligible. Based on these results, the 518 µm film was applied as the cooling layer in the Janus film. To further validate the role of TiO_2_, the solar reflectance (Figure S3) and MIR absorption (Figure S4) of the TiO_2_/PDMS film and a pure PDMS control were compared. Both films were cast on identical Cu substrates to maintain the same optical measurement configuration. The TiO_2_‐containing layer exhibited significantly higher solar reflectivity, particularly in the visible and near‐infrared regions, whereas both samples showed comparable MIR absorptivity. These results indicate that the high MIR absorptivity mainly originates from the intrinsic infrared absorption of the PDMS matrix, while TiO_2_ primarily contributes to enhanced solar reflectivity through strong light scattering. Thus, the improved cooling performance of the TiO_2_/PDMS layer is mainly attributed to enhanced solar rejection while maintaining the high MIR emissivity of the PDMS matrix. Control measurements without the Cu interlayer were further conducted to clarify the optical role of the Cu backing layer (Figure S5). The cooling‐side film without Cu showed a reduced maximum solar reflectivity of 90.40%, compared with 97% for the Cu‐backed TMP Janus film, confirming that the Cu interlayer contributes to solar‐light management by reflecting residual transmitted radiation. Meanwhile, the MIR reflectance remained very low without Cu, indicating that the high MIR absorptivity is primarily governed by the PDMS‐based functional layer rather than the Cu backing.

In order to investigate the radiative cooling and solar heating performance of the Janus film (10 cm × 10 cm) under cooling mode and heating mode respectively, the solar reflectivity and MIR absorptivity were presented in Figure 4a. With a high solar reflectivity of 97% and MIR absorptivity of 91%, the Janus film performed outstanding radiative cooling property under cooling mode. As shown in the thermal images in Figure 4b, the Janus film area delivers nearly the same color with the external heating temperature of 30°C and 40°C, indicating its outstanding high radiative heat loss. Even when the heating temperature was set as 50°C, the exterior surface temperature of the Janus film was much lower. This implies that the Janus film under cooling mode could provide desirable radiative cooling performance under daily solar irradiation. As shown in Figure 4c, the exterior surface temperature of the Janus film in heating mode exhibited rapid increase, rising from 25.4°C to 51.4°C within 100 s under 1 sun illumination, ultimately stabilizing at 65.4°C. In contrast, the cooling‐mode film maintained sub‐30°C temperatures regardless of irradiation intensity, demonstrating its capability to suppress solar heating even under intense sunlight.

**FIGURE 4:**
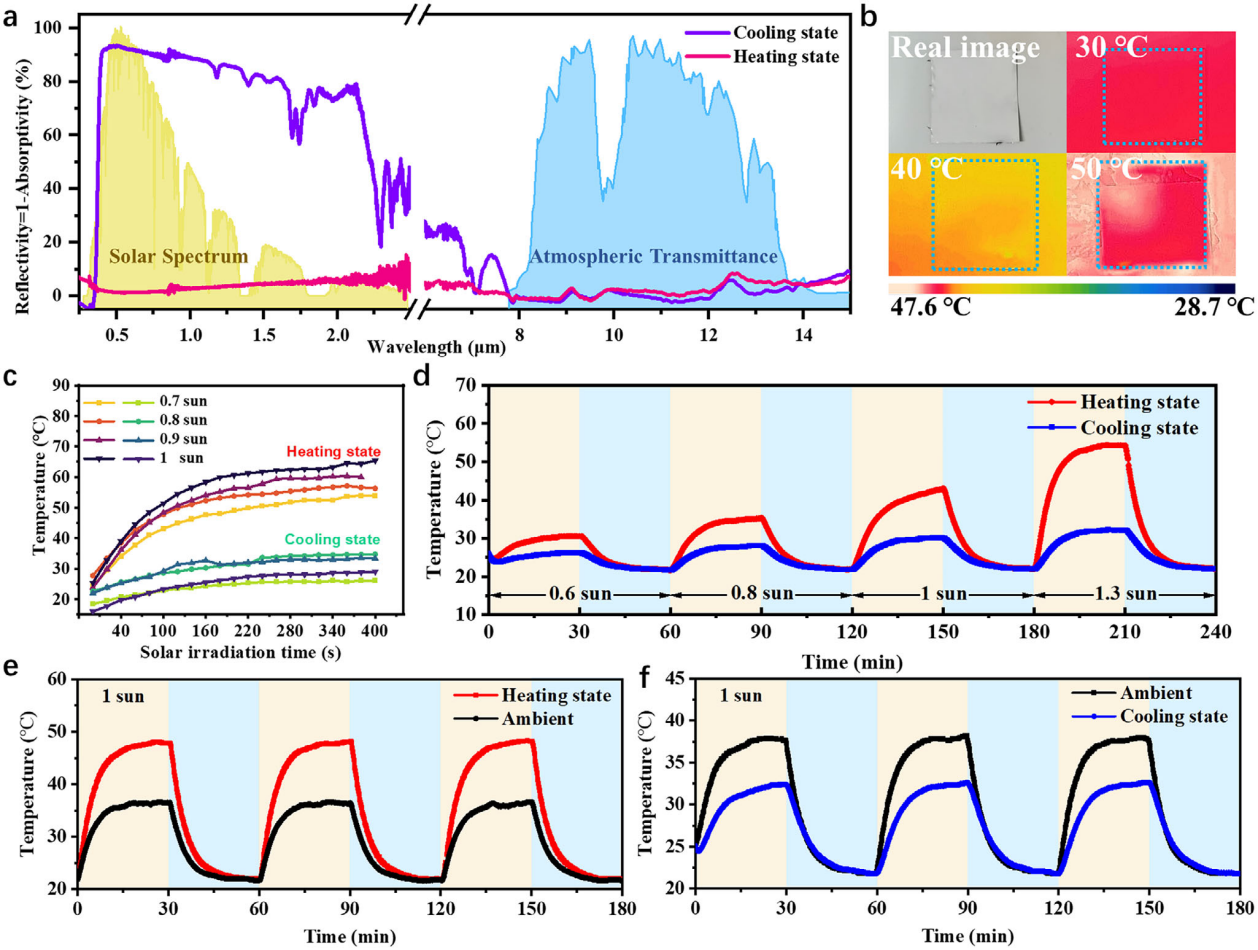
Optical and thermal characterization of the TMP Janus film. (a) Spectral reflectivity in solar (0.3–2.5 µm) and MIR (2.5–14 µm) ranges under cooling and heating modes. (b) Infrared thermal images of the TMP Janus film (10 × 10 cm^2^) with the TiO_2_/PDMS cooling layer facing upward at the heating stage temperature of 30°C, 40°C, and 50°C. (c) Exterior surface temperature of the film evolution under varying solar irradiation levels for both heating and cooling modes. (d) Temperature‐time curves profiles under different solar intensities showing increasing thermal response with irradiance. (e) Heating‐mode temperature evolution under 1 sun (1000 W/m^2^) illumination. (f) Cooling‐mode temperature evolution under 1 sun illumination.

To evaluate the practical thermoregulation capability of the TMP Janus film, sub‐ambient temperature changes were monitored in the air space behind the film under simulated solar irradiation. These measurements reflect the net thermal regulation effects under both heating and cooling modes Figure 4d shows the dynamic temperature response of the film under four different irradiance levels (0.6, 0.8, 1.0, and 1.3 sun illumination), representing varying outdoor solar intensities. In both heating and cooling modes, the sub‐ambient temperatures keep rising with irradiation time and approach equilibrium after approximately ten minutes. The temperature difference between the two modes also increases with irradiance, reaching 4.5°C, 7.3°C, 12.8°C, and 22.2°C at different irradiance levels. These results confirm enhanced photothermal conversion efficiency and adaptive thermal response across a wide range of environmental conditions.

Performance repeatability also plays a critical role for practical applications. Three consecutive heating‐mode tests were conducted under 1.0 sun illumination (Figure 4e). The temperature evolution profiles were highly consistent, with a maximum increase of 12.0°C. Under the same illumination condition, the film in the cooling mode reduced the rear‐side temperature by 6.0°C compared to ambient (Figure 4f). These results demonstrate that the TMP Janus film not only provides efficient solar heating and radiative cooling but also maintains stable performance across varying irradiation levels and repeated operation.

To evaluate the dual mode thermoregulation performance of the TMP Janus film in practical scenarios, outdoor testing was conducted on a building rooftop in Shenzhen, China (subtropical climate; November). A custom designed instrumentation system with distributed thermocouples and data loggers was utilized to continuously monitor the temperature profiles under dynamic solar irradiation, shown in Figure 5a. During clear‐sky duration, it achieved peak sub‐ambient cooling of 13.82°C under cooling mode and supra‐ambient heating of 7.21°C under heating mode, as documented in Figure 5b. It is worth noting that under non‐ideal weather conditions with variable solar irradiance, the film maintained consistent thermal regulation capability. The film consistently displays cooling effects of 17.89°C below ambient in windy conditions and 8.88°C during rainy days, and heating effects of 10.47°C and 6.46°C above ambient, respectively (Figure 5d,f). The observed differences in cooling and heating efficiency between windy and rainy conditions can be attributed to variations in air circulation, humidity, and illumination. Windy conditions enhance heat dissipation and evaporation, leading to more efficient cooling, while rainy days, with higher humidity and potentially lower solar irradiation, hinder both cooling and heating efficiency. These outdoor results under clear, windy and rainy conditions provide experimental evidence for the weather adaptability of the TMP Janus film. The cooling mode is expected to be more effective in hot and solar‐rich environments, where high solar reflectivity and MIR thermal emission can reduce heat gain, whereas the heating mode is more suitable for cold conditions with available solar irradiation. Under windy or rainy conditions, the thermoregulation effect may be weakened by enhanced convective heat exchange or reduced solar input, but the film still shows measurable temperature regulation capability.

**FIGURE 5:**
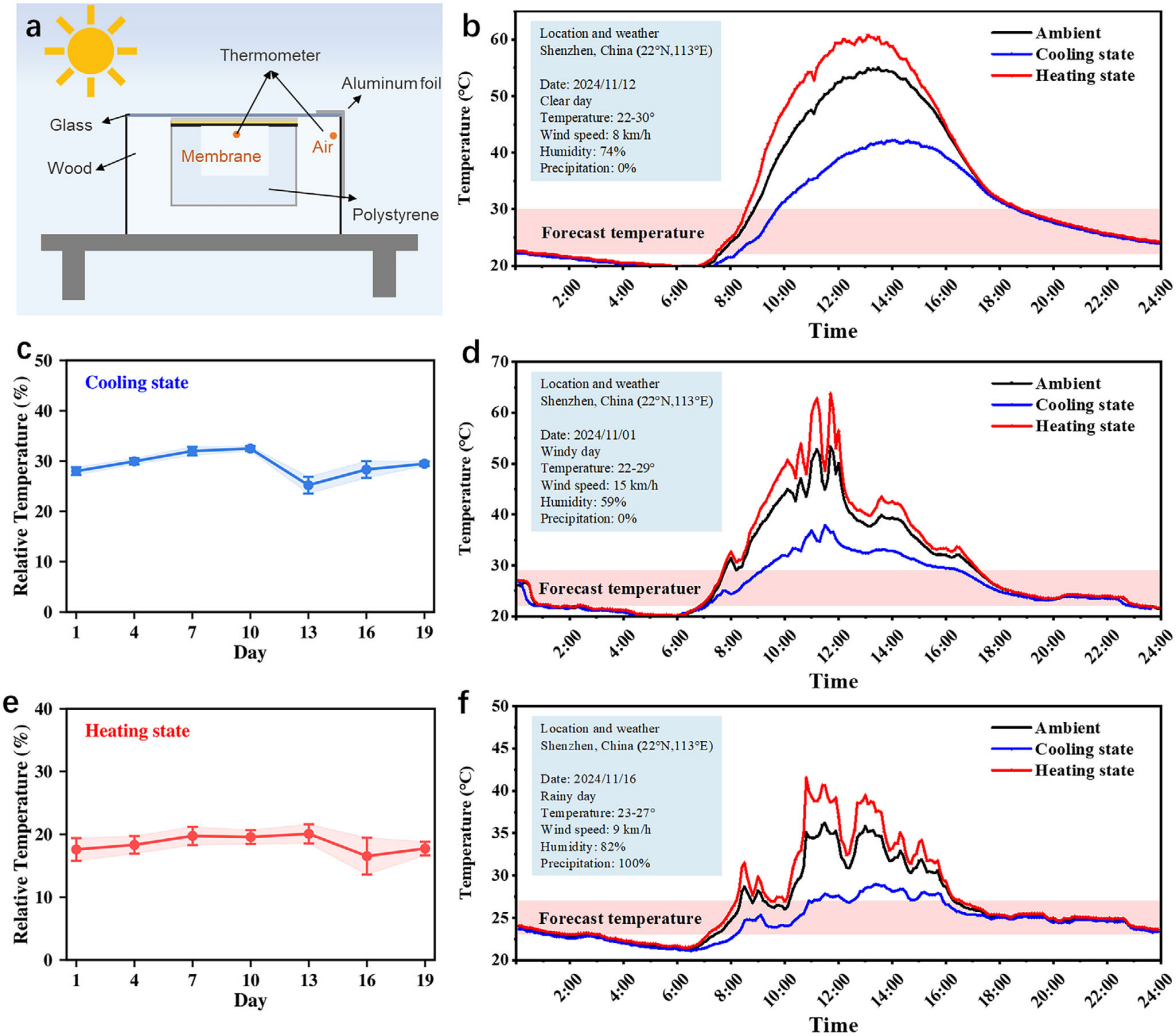
Thermoregulation performance of the TMP Janus film in outdoor scenarios. (a) Schematic diagram of the outdoor testing system. (b) Real‐time temperature profiles of the environment and tested samples recorded on a clear day (November 12, 2024). (c) Relative temperature reduction in cooling mode over 17 days. (d) Real‐time temperature profiles under windy conditions (November 1, 2024). (e) Relative temperature changes in heating mode over 17 days. (f) Real‐time temperature profiles under rainy conditions (November 16, 2024). Data in (c) and (e) are presented as mean ± standard deviation, calculated from temperature data recorded during 11:00–13:00 for each monitoring day.

Besides, to systematically evaluate the operational stability of the TMP film, a 17‐day continuous monitoring study was performed during peak solar hours from 11:00 to 13:00. The relative temperature difference was defined as the temperature difference between the thermoregulated space and the ambient environment, normalized by the ambient temperature. Under naturally fluctuating weather conditions, the cooling mode delivered a relative temperature difference of 25%–32%, while the heating mode showed a relative temperature difference of 16%–20%, as shown in Figure 5c,e, respectively. The data are presented as mean ± standard deviation, where the error bars represent the variation of relative temperature differences recorded within the monitoring period of each day. To further evaluate the durability of the TMP Janus film under practical environmental conditions, both the cooling‐mode and heating‐mode samples were exposed to outdoor solar irradiation for 20 days (Figure S6). After solar exposure, no obvious cracking, peeling, or delamination from the Cu foil was observed on either functional side, and the surface morphology remained nearly unchanged. These results indicate the long‐term reliability of the as‐prepared Janus film for thermal regulation. A summary of peak sub‐ambient cooling and above‐ambient heating values obtained under various conditions is provided in Table S1. These results reveal that the TMP Janus film is capable of passive, reversible, and high‐efficiency thermoregulation under realistic outdoor scenarios, which is promising for sustainable smart building applications.

To further demonstrate adaptive thermoregulation based on the as‐fabricated highly efficient TMP Janus film, a smart curtain system was designed for autonomous indoor temperature regulation. As illustrated in Figure 6a, the system consists of three major components: a TMP Janus curtain capable of dual‐mode thermoregulation through mechanical flipping, a motorized actuation mechanism with guiding tracks and pulleys for seamless mode switching, and a feedback control module featuring a thermometer, control circuitry, and Bluetooth communication for automated operation. The control module continuously monitors ambient temperature and triggers mode transitions when predefined thresholds are reached without manual operation, as shown in Figure 6b,c. Detailed photographs of the control circuitry and implementation are provided in Figure S7–S9. This feedback‐controlled switching process enables autonomous selection between cooling and heating modes while maintaining passive thermal regulation during operation.

**FIGURE 6:**
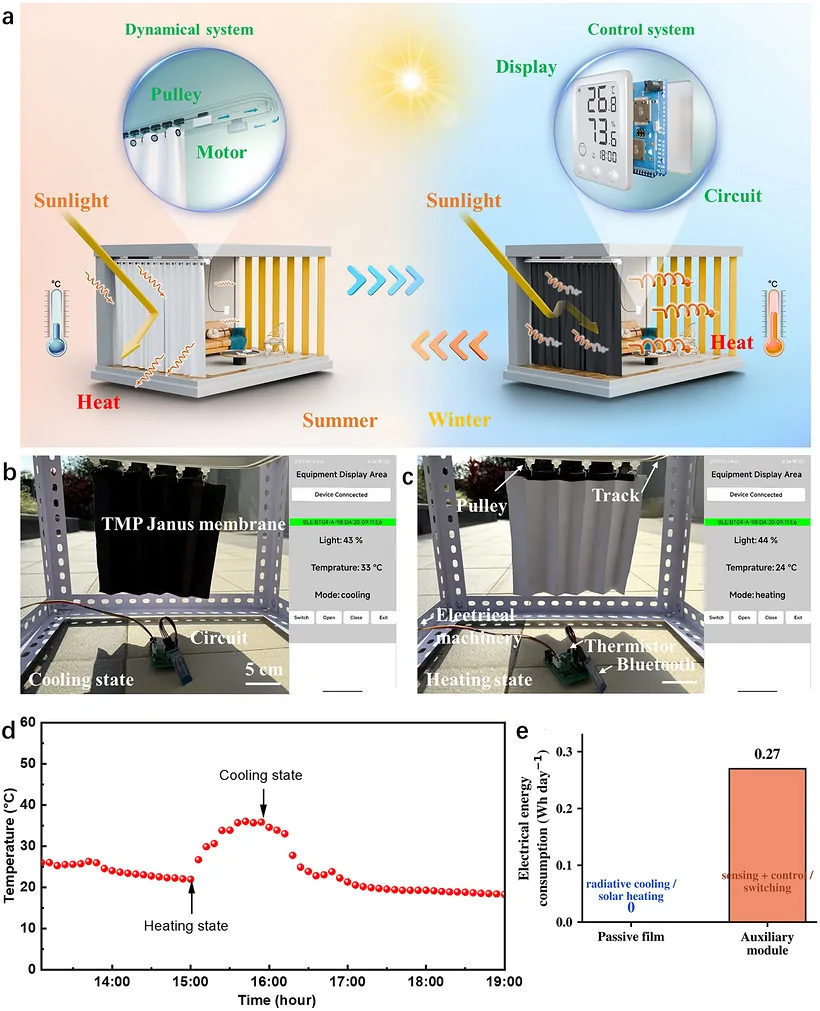
Proof‐of‐concept autonomous smart curtain system for adaptive indoor thermoregulation. (a) Schematic illustration of the smart curtain system comprising the TMP Janus film, a dynamic module, and a control system. Photograph and mobile app interface of the system in (b) cooling mode and (c) heating mode. (d) Real‐time temperature response profiles showing automatic mode switching triggered by preset thresholds. (e) Electrical energy consumption of the smart curtain prototype. The auxiliary energy consumption was calculated from the measured operating power and activation time of the sensing, control, and intermittent film‐orientation switching modules over one day.

The heating mode was set to activate at 22°C, while the cooling mode was triggered at 35°C, corresponding to the preset thermal comfort thresholds. The dynamic thermoregulation performance of the smart curtain system during an 8 h demonstration is shown in Figure 6d. Initially, the system operated in the cooling mode. When the ambient temperature decreased below 22°C, the control module automatically switched the curtain to the heating mode through film‐orientation switching. Conversely, when the temperature increased above 35°C, the system was reactivated into the cooling mode. The smart curtain prototype consumed only 0.27 Wh day^−^^1^ (Figure 6e), which was used for auxiliary temperature sensing, control, and intermittent film‐orientation switching. Since the TMP Janus film regulates heat exchange mainly through passive radiative cooling and solar heating, no direct electrical input is required for the thermoregulation process itself. Therefore, the proposed system provides a passive thermal‐management strategy that can reduce indoor thermal load and decrease reliance on active heating or cooling.

It should also be noted that the practical thermoregulation performance of the smart curtain will depend on the active film area, thermal load, solar irradiance, sky‐view factor, convective heat exchange, and building envelope conditions. In addition, the curtain‐based configuration inherently involves a trade‐off between thermoregulation and natural daylighting, because the film needs to cover the window to regulate solar heat gain and radiative exchange. Therefore, the current system is more suitable for scenarios where thermal regulation is prioritized, and future enhancement on film transparency or dynamically deployable designs may further improve the balance between temperature regulation and daylight availability.

## 3 Conclusion

The development of advanced thermoregulation systems with the capability to dynamically adapt to environmental fluctuations remains a critical unmet need in sustainable architecture. This work proposes a multi‐dimensional TiO_2_/MXene/PDMS Janus film that is capable of dual‐mode radiative cooling and solar heating. The rationally designed architecture delivers a 97% solar reflectivity for cooling and a 91% solar absorption for heating. Outdoor evaluations deliver ultra‐wide range temperature regulation with sub‐ambient cooling of 17.89°C and above‐ambient heating of 13.82°C. The as‐prepared film was further developed into an autonomous smart curtain for practical temperature regulation, which consumes merely 0.27 Wh daily for auxiliary motorized switching, highlighting its potential to reduce reliance on active HVAC systems. This transformative platform can serve as a highly promising solution for sustainable and effective thermoregulation.

## 4 Experimental Section

### 4.1 Materials

PDMS precursor used in our experiments was Sylgard 184 (Dow Corning Co.). TiO_2_ powders were purchased from Shanghai Macklin Biochemical Co., Ltd., China. MXene were provided by 11 Technology Co., Ltd., China.

### 4.2 Preparation of a Janus Thermoregulation Film

The TMP Janus film with a “sandwich” structure was obtained by two step casting method. First, TiO_2_ powders were dissolved in PDMS base by intense mechanical agitation to a uniform emulsion state. And then curing agent was added in, forming the TiO_2_‐PDMS‐curing agent precursor solution in an 11:10:1 mass ratio followed by intense stirring mixing for 30 mins. Additionally, the Cu foil with 20 µm thickness was fixed on a glass flake and then treated with ozone to remove impurities from the surface making the TiO_2_/PDMS film more tightly covered. Afterward, the TiO_2_‐PDMS‐curing agent precursor solution was poured on the Cu foil placed on the homogenizer. By increasing the speed and time of spin coating, TiO_2_/PDMS films with different thicknesses were fabricated. Then the TiO_2_/PDMS film was vacuumed for over 60 min at room temperature followed by heated up to 90°C and the duration of heat treatment was 2 h. Next, it slowly cooled down to the room temperature and the TiO_2_/PDMS film was solidified on the Cu foil.

Then the TiO_2_/PDMS film with Cu foil was fixed on the glass flake with Cu foil facing upward followed by peeled off and flipped. Next, the PDMS base and MXene with a mass ratio of 1:0.3 was mixed by severe continuous stirring for 3 h. Then, a curing agent with 1/10 weight of PDMS base was added to the mixture, accompanied by stirring of 30 mins. Subsequently, the prefabricated TiO_2_/PDMS film with Cu foil fixed on the glass flake with Cu foil facing upward was treated with ozone to make MXene/PDMS film more tightly covered. The obtained homogeneous MXene‐PDMS‐curing agent emulsion was poured on the Cu foil surface of the TiO_2_/PDMS film using a film applicator. After vacuumed for over 60 min at room temperature and heated treatment at 90°C for 2 h, the MXene/PDMS film was cured on the Cu foil of the TiO_2_/PDMS. After peeling off from the glass flake, a free‐standing TMP Janus film was obtained.

### 4.3 Preparation of Control Samples

For the optical comparison experiments, several control samples were prepared using the same Cu‐backed configuration as the TMP Janus film. To prepare the pure PDMS blank layer, PDMS base and curing agent were mixed at a mass ratio of 10:1 and stirred for 30 min, followed by vacuum degassing for over 60 min. A Cu foil with a thickness of 20 µm was fixed on a glass substrate and treated with ozone under the same conditions as those used for the Janus film. The pure PDMS precursor was then cast onto the Cu foil and cured at 90°C for 2 h. The thickness of the pure PDMS layer was controlled to be comparable to that of the TiO_2_/PDMS cooling layer.

For the heating‐layer control sample, the MXene‐free PDMS/Cu sample was prepared by casting pure PDMS precursor onto the ozone‐treated Cu foil, followed by the same degassing and curing process. The MXene/PDMS/Cu sample was prepared by mixing PDMS base and MXene at a mass ratio of 1:0.3 under continuous stirring for 3 h, followed by the addition of curing agent at one‐tenth of the PDMS base mass. After further stirring for 30 min and vacuum degassing, the MXene/PDMS precursor was cast onto the Cu foil using a film applicator and cured at 90°C for 2 h. All control samples were prepared with the same Cu foil thickness, curing conditions, and measurement geometry as the corresponding layers in the TMP Janus film.

### 4.4 Characterization

The morphology and structure of the Janus film were analyzed by a field‐emission scanning electron microscopy (Zeiss Sigma 500). Reflectance measurements in the wavelength range of 0.3–2.5 µm were recorded using a Lambda 750s UV–vis–NIR spectrophotometer equipped with an integrating sphere (PerkinElmer Inc.). The transmittance and reflectance in the wavelength range of 2.5–14 µm were conducted on Fourier transform infrared spectrometer (Vertex 70v, Bruker, with a gold integrating sphere (Model 4P‐GPS‐020‐SL, Labsphere). The temperature distribution images were obtained in real time using an infrared camera (FLIR ONE Pro) with a working distance of approximately 20 cm. Simulation of Sunlight Exposure Experiment Using a Solar Simulator (Sun 3000, Abet Technologies).

For optical characterization, all Cu‐backed samples were measured with the functional polymer layer facing the incident beam and the Cu foil positioned on the backside, consistent with the working configuration of the Janus film. The same Cu foil thickness and measurement geometry were used for all control and experimental samples. Therefore, the optical contribution from the Cu layer was kept constant in comparative measurements. The differences between the pure PDMS/Cu and TiO_2_/PDMS/Cu samples were used to evaluate the role of TiO_2_ in enhancing solar reflection, while the differences between the PDMS/Cu and MXene/PDMS/Cu samples were used to assess the role of MXene in solar absorption. The reported absorptivity was calculated as A = 1 – R − T. Since the Cu‐backed samples were optically opaque, the transmittance was negligible, and the absorptivity was approximated as A = 1 − R. For the control measurements without the Cu interlayer, the corresponding TiO_2_/PDMS cooling‐side film and MXene/PDMS heating‐side film were prepared using the same precursor formulations and curing conditions as those used for the TMP Janus film, except that the Cu foil was not introduced. The solar reflectivity was obtained from the UV–vis–NIR reflectance spectra in the wavelength range of 0.3–2.5 µm. For the MIR region, both reflectance and transmittance were measured, and the MIR absorptivity was calculated according to A = 1 − R − T.

### 4.5 Outdoor Thermal Measurements

The main component of the outdoor thermal measure equipment is an aluminum foil‐coated wooden frame chamber with a polystyrene foam block. The wooden frame was 20 cm high and 30 × 30 cm wide while the polystyrene foam block was 15 cm high and 20 × 20 cm wide. The polystyrene foam block was embedded in the wooden frame chamber without any gaps. The wooden frame was covered by an aluminum foil to prevent heating from sunlight. The foam was used to reduce convective heat loss to the Janus film and improve thermal isolation. The fabricated film to be measured was placed in the 10 × 10 cm square groove in the center of the foam block with contacting edges as small as possible to minimize the heat exchange. The entire device is covered with glass sheets to simulate the configuration of curtains. To measure the real‐time temperature change of the Janus film and the ambient air near the measure equipment, two K‐type thermocouples connected to temperature recorders were placed in the equipment and recorded the data at intervals of 6 mins utilizing a data logger (HOBO UX120‐014 M, Onset Computer Corp.).

### 4.6 Principle of Radiative Cooling

Considering blackbody radiation, thermal exchange with the atmosphere, the solar irradiation, and heat exchange channels through conduction and convection, the total cooling power *P*_cool_(*T*) of the TMP Janus film under cooling mode can be defined as:

(1) $$P_{\mathrm{cool}}\;\left(T\right)=P_{\mathrm{rad}}\;\left(T\right)-P_{\mathrm{atm}}\left(T_{\mathrm{amb}}\right)-P_{\mathrm{sun}}-P_{\mathrm{cc}}\left(T,T_{\mathrm{amb}}\right)$$

where *P*_rad_(*T*) is the power radiated by the film, *P*_atm_(*T*_amb_) is the power of the incident atmospheric radiation by atmospheric heat exchange outside the atmospheric window (AW) region, *P*_sun_ is the absorbed incident power from the sun, *P*_cc_(*T, T*_amb_) is the conduction and convection power, *T* is the temperature of the radiative cooling device, and *T*_amb_ is ambient temperature.

The radiation power *P*_rad_(*T*) is the power radiated by the film which is expresses as:

(2) $$P_{\mathrm{rad}}\;\left(T\right)=A\int d\Omega \cos\theta \int_{0}^{\infty }d\lambda I_{\mathrm{BB}}\left(T,\lambda \right)\varepsilon \left(\lambda ,\theta \right)$$

where *A*, *λ*, *θ* and $\int d\Omega =2\pi \int_{0}^{\pi /2}\sin\theta d\theta$ are the surface area of the sample, the wavelength, polar angle, and angular integral over a hemisphere, respectively.

The blackbody specific intensity *I*_BB_(*T, λ*) at *T* is

(3) $$I_{\mathrm{BB}}\;\left(T,\lambda \right)=\frac{2hc^{2}}{\lambda^{5}}\;\frac{1}{e^{\frac{\mathrm{hc}}{\left(\lambda k_{B}T\right)}}-1}$$

where *h* is Plank's constant, *c* is the speed of light in vacuum and *λ* is the wavelength.

The power of the incident atmospheric radiation *P*_atm_(*T*_amb_) by atmospheric heat exchange outside the AW region is:

(4) $$P_{\mathrm{atm}}\;\left(T_{\mathrm{amb}}\right)=\int \int_{0}^{\infty }I_{\mathrm{BB}}\left(T_{\mathrm{amb}},\lambda \right)\varepsilon \left(\lambda ,\theta \right)\varepsilon_{\mathrm{atm}}\left(\lambda ,\theta \right)d\lambda \cos\theta d\Omega$$

where ε_atm_ (λ,θ) =  1 − *t*(λ)^1/cos θ^ and *t*(*λ*) are the atmospheric emissivity and the atmospheric transmittance in the zenith direction, respectively.

The absorbed incident power from the sun *P*_sun_ can be expressed as:

(5) $$P_{\mathrm{sun}}=A\int_{0}^{\infty }d\lambda \varepsilon \left(\lambda ,\theta_{sun}\right)I_{AM1.5}\left(\lambda \right)$$

where *I*_AM1.5_ is the solar illumination intensity.

According to Newton's law, the conduction and convection power *P*_cc_(*T, T*_amb_) is:

(6) $$P_{\mathrm{cc}}\;\left(T,T_{amb}\right)=Ah_{\mathrm{cc}}\;\left(T_{\mathrm{amb}}-T\right)$$

where h_cc_ the coefficient of the non‐radiative transfer mechanisms. Kirchhoff's law states that emissivity ε is equal to the absorptivity [56], so it can be used interchangeably.

For outdoor passive radiative cooling films, the P_sun_ in the region 300–2500 nm should be minimized and the Prad in the AW must be maximized so that most of the incident solar irradiance can be reflected and more heat can be radiated into space, which results in a reduction of the temperature under the PRC film.

## Author Contributions

**Y.S**. (co‐first author): Conceptualization, Data analysis, Writing – original draft, Methodology, Experimentation design, Sample preparation, Experimental measurement. **Y.F**. (co‐first author): Conceptualization, Data analysis, Writing – original draft, Methodology, Experimentation design, Sample preparation, Experimental measurement. **X.Y**.: Circuit design, system integration. **Y.G**.: Data Curation, Writing – Original Draft. **Y.L**.: Methodology, Supervision. **D.L**.: Conceptualization, Funding Acquisition, Resources, Supervision, Writing – Review & Editing. **Y.L**. (Corresponding Author): Conceptualization, Funding Acquisition, Resources, Supervision, Writing – Review & Editing.

## Conflicts of Interest

The authors declare that they have no known competing financial interests or personal relationships that could have appeared to influence the work reported in this paper.

## Supporting information




**Supporting file**: advs76462‐sup‐0001‐SuppMat.docx

## Data Availability

The data that support the findings of this study are available from the corresponding author, YJ. Lin, upon reasonable request.
