# Supplementary material for: Adaptive and Highly Efficient Thermoregulation Based on Multi‐Dimensional Janus Film
Source: Adv Sci (Weinh). 2026 Jul 9;13(56):e76462. doi: 10.1002/advs.76462 (PMC13348343; doi:10.1002/advs.76462)
Supplement: Supplementary file 1 [file ADVS-13-e76462-s001.docx]

Supplementary Materials

Adaptive and Highly Efficient Thermoregulation based on Multi-dimensional Janus Film

*Yuqing Shi^#^, Yuting Fu^#^, Xiong Yu, Yuao Guo, Yanjun Liu, Dan Luo^*^, Yuanjing Lin ^*^*

Y. Shi, X. Yu, Y. Lin

School of Microelectronics, Southern University of Science and Technology, Shenzhen 518055, China.

E-mail: [linyj2020@sustech.edu.cn](mailto:linyj2020@sustech.edu.cn).

Y. Fu, Y. Guo, Y. Liu, D. Luo

Department of Electrical and Electronic Engineering, Southern University of Science and Technology, Shenzhen 518055, China.

E-mail: [luod@sustech.edu.cn](mailto:luod@sustech.edu.cn).

Y. Lin

SUSTech Energy Institute for Carbon Neutrality, Southern University of Science and Technology, Shenzhen, 518055, China

State Key Laboratory of Quantum Functional Materials, Southern University of Science and Technology, Shenzhen 518055, China.

The Key Laboratory of the Third Generation Semi-conductor, Southern University of Science and Technology, Shenzhen, 518055, China.

Y. Shi

Department of Applied Biology and Chemical Technology, The Hong Kong Polytechnic University, 999077, Hong Kong Special Administrative Region of China.

**Figure S1.**Simultaneous monitoring of heat flow (via differential scanning calorimetry, DSC) and mass variation (via thermogravimetric analysis, TGA) for MXene films under controlled thermal cycling.

**Figure S2.** SEM images of the TiO2 microspheres with diameter of 2 μm.

1 μm

**Figure S3.** Solar reflectivity of TiO₂-doped PDMS cooling layer of the Janus film and blank layer.

**Figure S4.** MIR absorptivity of TiO₂-doped PDMS cooling layer of the Janus film and blank layer.

**Figure S5.** Spectral reflectivity in solar (0.3-2.5 μm) and MIR (2.5-14 μm) ranges under cooling and heating modes without Cu interlayer.

**Figure S6.** Outdoor solar-exposure stability of the TMP Janus film.

**Figure S7.** The digital picture of the control system, including motor, circuit and Bluetooth.

**Figure S8.** Circuit design of the control system. (1) MCU; (2) Motor interface; (3) Power interface; (4) Exclusion; (5) Crystal oscillator; (6) Switch;(7) Motor control module; (8) Bluetooth interface; (9) Thermistor.

**Figure S9.** The circuit schematic of the control system.

**Table S1.** Comparison of films with dual functions of radiative cooling and solar heating

| **Materials / system** | **Switching mechanism** | **Solar reflectivity at cooling mode** | **MIR absorptivity at cooling mode** | **Solar reflectivity at heating mode** | **MIR absorptivity at heating mode** | **Sub-ambient temperature drop** | **Above-ambient temperature rise** | **Device integration / validation** | **Ref.** |
| --- | --- | --- | --- | --- | --- | --- | --- | --- | --- |
| Dynamic cavitation of silicone coatings based on porous PDMS and Carbon Black | Strain-induced dynamic cavitation | 93% | 94% | 5% | -- | 5°C | 28°C | Stretchable coating-level thermal regulation | [1] |
| Thermochromic smart window based on hydroxypropyl cellulose hydrogel. | Thermochromic transition | 92.2% | 87% | 28.4% | 87% | 5.4°C | 4.5°C | Smart window device | [2] |
| Smart window based on thermotropic hydrogel | Thermotropic transition | 65.19% | -- | 11.32% | -- | 10.8°C | -- | Smart window device | [3] |
| Thermochromic smart window based on VO2 | Thermochromic phase transition | 26.1% | 61% | 27.8% | 21% | -- | -- | Smart window device | [4] |
| Janus membrane based on boron nitride-polydimethylsiloxane and graphene nanoplatelet-polydimethylsiloxane nanofiber | Janus orientation switching | 93.7% | 96.2% | 7.1% | -- | 4°C | 48.9°C | Janus membrane-level dual-mode thermal regulation | [5] |
| Janus textile based on AgNWs and MXene | Janus orientation switching | 40.2% | 47.3% | 12.4% | 18.9% | -1.7°C | 16°C | Janus textile-level thermal regulation | [6] |
| Shape-morphing porous TPU integrated with photothermal MXene layer | Programmable shape morphing / folding-angle modulation | 95.1% | 88.5% | Folding-angle-dependent | -- | -- | -- | Multimode adaptive thermoregulation prototype | [7] |
| TMP Janus film | Temperature-feedback-controlled orientation switching | 97% | 91% | 9% | -- | 17.89°C | 13.82°C | Smart curtain with outdoor validation | This work |

**Reference:**

[1]	H. Zhao, Q. Sun, J. Zhou, X. Deng, J. Cui, Advanced Materials 2020, 32, 2000870.

[2]	S. Wang, Y. Zhou, T. Jiang, R. Yang, G. Tan, Y. Long, Nano Energy 2021, 89, 106440.

[3]	Y. Niu, Y. Zhou, D. Du, X. Ouyang, Z. Yang, W. Lan, F. Fan, S. Zhao, Y. Liu, S. Chen, Advanced Science 2022, 9, 2105184.

[4]	S. Wang, T. Jiang, Y. Meng, R. Yang, G. Tan, Y. Long, Science 2021, 374, 1501.

[5]	Y. Jung, S. Jeong, J. Ahn, J. Lee, S. H. Ko, Small 2024, 20, 2304338.

[6]	L. Tang, B. Lyu, D. Gao, Z. Jia, Y. Fu, J. Ma, Small 2024, 20, 2308194.

[7] Y. Choi, S. Tang, H. Woo, K. Zhou, P. Karua, L. Cai. Advanced Engineering Materials 2025; 27:e202501580.
